# Myelitis Following COVID-19 Illness

**DOI:** 10.7759/cureus.28134

**Published:** 2022-08-18

**Authors:** Nihal Gulati, Saniya Kapila, Lucky Bhalla Sehgal, Vineet Sehgal, Priyal LNU

**Affiliations:** 1 Medicine, Navpreet Hospital, Amritsar, IND; 2 Medicine, Fortis Escorts Hospital, Amritsar, IND; 3 Paediatrics, Sehgal's Neuro and Child Care Centre, Amritsar, IND; 4 Neurology, Amandeep Medicity, Amritsar, IND; 5 Neurology, Lady Hardinge Medical College, New Delhi, IND

**Keywords:** mri spine, para-infectious covid myelitis, letm, myelitis, covid-19

## Abstract

COVID-19 occurs due to infection by the SARS-CoV-2 virus (severe acute respiratory syndrome coronavirus 2), which has caused havoc globally. It presents with a wide range of symptoms, mainly respiratory symptoms, but with time various neurological manifestations of the disease have also been noted, like myelitis. This case report aims to shed light on COVID-19-associated myelitis so that potential neurological complications of COVID-19 can be identified and treated timely. We report a case of a 41-year-old male who presented with weakness of all limbs with urinary complaints. He also had a cough and sore throat for the past few days. The MRI scan of the spine showed long segment myelitis in the cervical cord extending from the cervicomedullary junction to the upper end of the C4 vertebral body. COVID-19 myelitis is a rare but severe complication of COVID-19 infection and needs to be discussed.

## Introduction

COVID-19 began in Wuhan, China, and spread worldwide to become a pandemic. Symptoms of COVID-19 infection include fever, sore throat, cough, fatigue, breathlessness, loss of smell and taste, etc. However, a large number of people remain asymptomatic [1]. Although this virus mainly affects the lungs, many extrapulmonary manifestations have been seen, like neurological complications, myocardial dysfunction, acute kidney injury, hepatocellular injury, dermatological changes, etc. [2]. Neurological sequelae of the disease include COVID-19-associated myelitis, Guillain-Barre syndrome, a demyelination disorder associated with anti-MOG (myelin oligodendrocyte glycoprotein) antibodies, encephalitis, acute disseminated encephalomyelitis, seizures, epidural abscess, cranial neuropathies, etc. [3]. Myelitis refers to spinal cord inflammation, leading to autonomic, sensory, and motor dysfunction in the body.

## Case presentation

A 41-year-old male presented with complaints of weakness of all limbs, difficulty passing urine, and mild cough and sore throat for the last five days. There was no history of shortness of breath, chest pain, fever, loss of taste or smell, trauma, and recent vaccination.

On examination, his Glasgow Coma Scale (GCS) was 15/15, with no visible atrophy or fasciculations, and tone was increased in lower limbs but normal in upper limbs. Power in upper limbs was grade 4 proximally and grade 3 distally. In lower limbs, power was grade 3 proximally and distally. All the deep tendon reflexes were brisk and with extensor plantar response. The sensory loss was evident below the C2 level in all modalities. 

**Figure 1:**
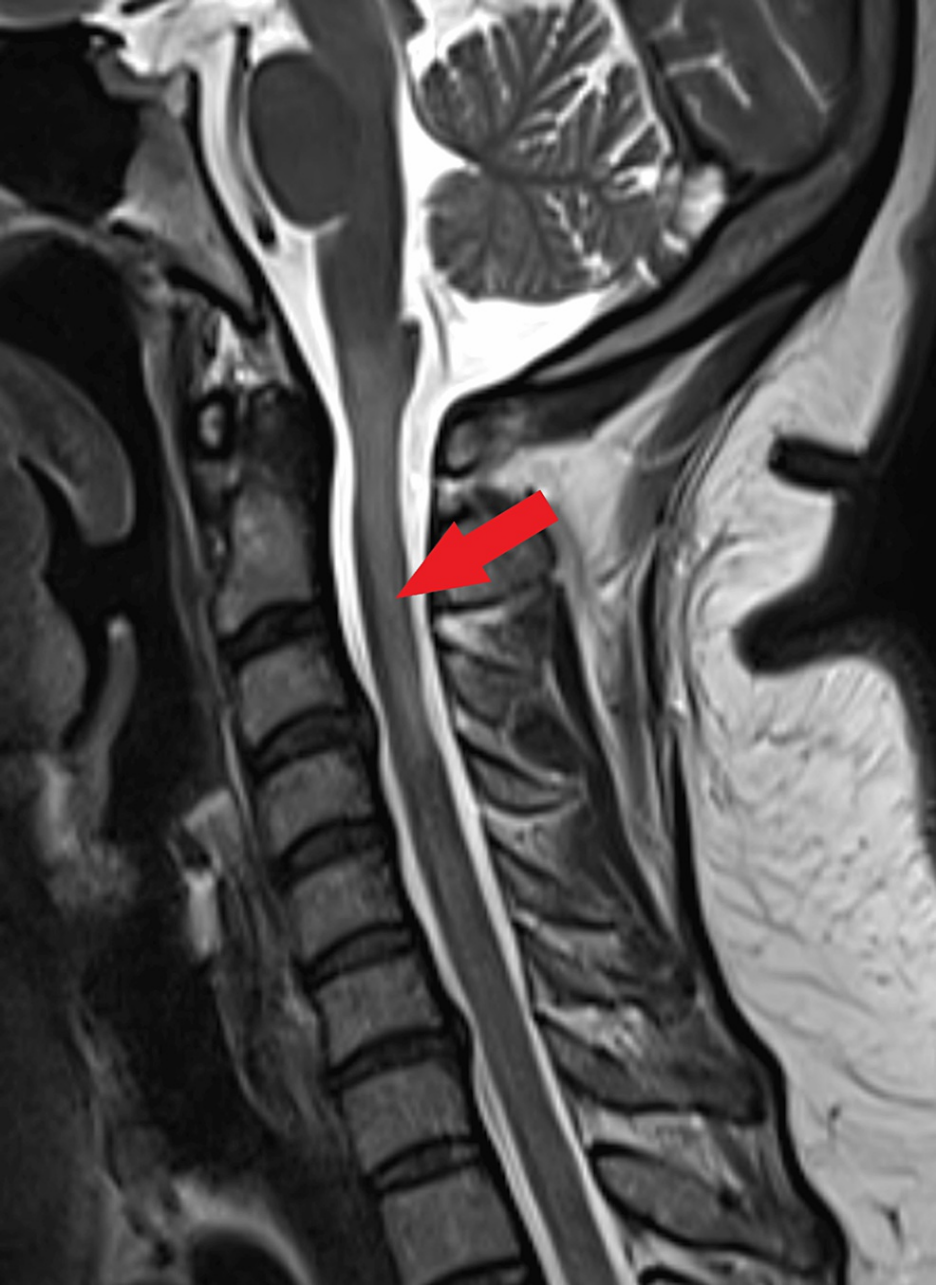
Long segment hyperintense signal in the cervical cord extending from the cervicomedullary junction to upper end of C4 vertebral body on T2-weighted sagittal image.

**Figure 2:**
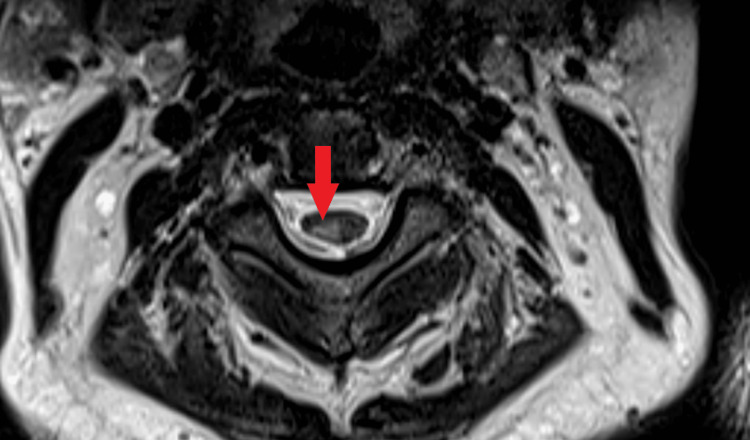
T2-weighted axial image showing hyperintense signal in the cervical cord on the right side.

The patient was admitted for evaluation of his complaints. Chest X-Ray and CT scan of the chest were normal. However, the COVID-19 polymerase chain reaction (PCR) test of the patient came out to be positive and the patient was diagnosed as a case of mild COVID-19 illness. Blood investigations were normal except for raised C-reactive protein (CRP) levels (26 mg/l). The MRI scan of the brain showed no abnormality. However, MRI with contrast of the whole spine showed a long segment hyperintense lesion in the cervical cord extending from the cervicomedullary junction to the upper end of the C4 vertebral body in the sagittal view in T2-weighted (T2W) images (Figure 1). The axial view of the cervical spinal cord showed T2 hyperintense lesions on the right side (Figure 2). These hyperintense lesions were not contrast-enhancing lesions. The cerebrospinal fluid (CSF) evaluation showed raised lymphocytes (88 cells/mm^3^), elevated proteins (65 mg/dl), and normal glucose levels. The CSF examination was negative for oligoclonal bands, malignant cells, all stains, pan neurotropic virus panel, and COVID-19 PCR. Blood investigations for antibodies like c-ANCA (anti-neutrophil cytoplasmic antibodies), p-ANCA, ANA (antinuclear antibody), MOG (myelin oligodendrocyte glycoprotein) antibodies, and aquaporin-4 antibodies were negative.

Thus, the patient was clinically suspected to be a case of para-infectious COVID-19 myelitis [4]. He was given intravenous steroids (methylprednisolone 1 gram daily) for five days, followed by tapering dosages of oral steroids for four weeks. He started showing improvement, and after about five weeks of initiation of treatment, he could walk without support and void urine without any problem.

## Discussion

Transverse myelitis (spinal cord inflammation) can cause symptoms such as weakness in limbs, sensory loss, urinary or bowel disturbances, and sexual dysfunction [5]. The cause of transverse myelitis can be unknown (idiopathic), associated with autoimmune diseases such as multiple sclerosis, systemic lupus erythematous, anti-MOG related disease, neuromyelitis optica, or after bacterial, viral, or fungal infection, paraneoplastic syndrome, or post-vaccination [5]. MRI scan of the spine plays a crucial role in diagnosing myelitis and demarcating the involved segments [6]. Based on the MRI findings, myelitis can be called short segment myelitis (when the length of T2 hyperintense lesions in sagittal MRI images is less than three vertebral segments) or longitudinally extensive transverse myelitis (LETM), when the lesions are equal to or more than three vertebral segments long [7].

COVID-19 infection has been associated with myelitis, and the first case was reported from Wuhan in a 66-year-old man who developed paraparesis about one week after the onset of fever [8]. A few other cases from different parts of the world have been reported since then. Sarma and Bilello, in 2020, reported a case of a 28-year-old woman (known case of hypothyroidism) who had tested positive for COVID-19 infection and later developed paresthesia in her lower limbs [9]. It progressed to loss of sensation in upper and lower limbs, numbness of the tip of the tongue, and urinary retention. MRI spine showed LETM. Similarly, Valiuddin H. et al. reported a case of a 61-year-old female who presented with generalized body weakness, tingling in her hands and feet, weakness in both lower limbs, constipation, and difficulty voiding [10]. One week before these symptoms, she had rhinorrhoea and chills. Investigations revealed positive COVID-19 PCR, and the MRI of the spine showed LETM. Likewise, Ali L et al. reported two cases of COVID-19-related myelitis, presenting after four to five days of COVID-19 illness symptoms such as fever, sore throat and headache etc. [11]. COVID-19 illness is associated with both short-segment and long-segment myelitis [12, 13]. 

Our patient presented with weakness of both upper and lower limbs, difficulty passing urine, sore throat, and cough. We have diagnosed this as a case of probable COVID-19 myelitis given signs and symptoms of COVID-19, positive COVID-19 PCR, CSF findings, and absence of any other explanatory cause. Although many reported cases have symptoms of myelitis one to six weeks after COVID-19 illness, the infection can be more recent in some cases, like our patient [14]. Intravenous methylprednisolone and supportive management for COVID-19 help improve symptoms in most cases.

The exact etiopathogenesis of COVID-19 myelitis is unknown, but three main mechanisms have been proposed. First is direct neuronal injury by coronavirus [14]. Second, the virus binds strongly to angiotensin-converting enzyme 2 (ACE2) receptors in the heart, lungs, CNS, and skeletal muscles. It causes activation of ACE2 receptors in the CNS and triggers an inflammatory response in the body. This leads to a cytokine storm (increased IL-6, IL-1, and tumour necrosis factor (TNF) alpha levels), leading to glial cell activation and demyelination [14,15]. The third mechanism is seen in post-infectious patients, where damage to the CNS is caused by molecular mimicry [14]. Since COVID-19 symptoms were present in our patient along with the positive COVID-19 PCR during myelitis, the most likely mechanism seems to be either the first or second. It is to be noted that CSF COVID-19 PCR was negative in our case.

Corticosteroids serve as the first line of treatment for COVID-19 myelitis [16]. However, if the patient does not improve, intravenous immunoglobulins (IVIG) or plasmapheresis can be helpful [13,17].

## Conclusions

Myelitis is a rare but important neurological manifestation of COVID-19. The clinical picture, CSF analysis, serological analysis, and radiological investigations such as MRI of the spine help to rule out the differential diagnoses. Corticosteroids serve as the mainstay of treatment.

It is essential to understand that COVID-19 myelitis if identified quickly and managed without delay can result in a better prognosis in patients.

## References

[1] (2022). Coronavirus disease 2019 (COVID-19). https://www.mayoclinic.org/diseases-conditions/coronavirus/symptoms-causes/syc-20479963.

[2] Gupta A, Madhavan MV, Sehgal K (2020). Extrapulmonary manifestations of COVID-19. Nat Med.

[3] Niazkar HR, Zibaee B, Nasimi A, Bahri N (2020). The neurological manifestations of COVID-19: a review article. Neurol Sci.

[4] Jagadeesan S, Kamra N, Meena RC, Patel P (2021). Parainfectious longitudinal extensive transverse myelitis (LETM) post-COVID-19 - a rare report. Neurol India.

[5] (2022). Transverse Myelitis (TM). https://my.clevelandclinic.org/health/diseases/8980-transverse-myelitis.

[6] Chow CC, Magnussen J, Ip J, Su Y (2020). Acute transverse myelitis in COVID-19 infection. BMJ Case Rep.

[7] Nightingale H, Witherick J, Wilkins A (2011). Diagnosis of longitudinally extensive transverse myelitis. BMJ Case Rep.

[8] Chakraborty U, Chandra A, Ray AK, Biswas P (2020). COVID-19-associated acute transverse myelitis: a rare entity. BMJ Case Rep.

[9] Sarma D, Bilello LA (2020). A case report of acute transverse myelitis following novel coronavirus infection. Clin Pract Cases Emerg Med.

[10] Valiuddin H, Skwirsk B, Paz-Arabo P (2020). Acute transverse myelitis associated with SARS-CoV-2: a case-report. Brain Behav Immun Health.

[11] Ali L, Mohammed I, Zada Y, Salem H, Iqrar A (2021). COVID-19-associated acute transverse myelitis: a case series of a rare neurologic condition. Cureus.

[12] Sehgal V, Arora S, Bansal P, Kapila S, Bedi GS, Priyal Priyal (2022). COVID-19 associated myelitis: case series. Int J Adv Med.

[13] Schulte EC, Hauer L, Kunz AB, Sellner J (2021). Systematic review of cases of acute myelitis in individuals with COVID-19. Eur J Neurol.

[14] Román GC, Gracia F, Torres A, Palacios A, Gracia K, Harris D (2021). Acute transverse myelitis (ATM): clinical review of 43 patients with COVID-19-associated ATM and 3 post-vaccination ATM serious adverse events with the ChAdOx1 nCoV-19 vaccine (AZD1222). Front Immunol.

[15] Wu Y, Xu X, Chen Z (2020). Nervous system involvement after infection with COVID-19 and other coronaviruses. Brain Behav Immun.

[16] Ahmad SA, Salih KH, Ahmed SF (2021). Post COVID-19 transverse myelitis; a case report with review of literature. Ann Med Surg (Lond).

[17] Baghbanian SM, Namazi F (2021). Post COVID-19 longitudinally extensive transverse myelitis (LETM)-a case report. Acta Neurol Belg.

